# Identifying novel genetic loci associated with polycystic ovary syndrome based on its shared genetic architecture with type 2 diabetes

**DOI:** 10.3389/fgene.2022.905716

**Published:** 2022-08-29

**Authors:** Xiaoyi Li, Han Xiao, Yujia Ma, Zechen Zhou, Dafang Chen

**Affiliations:** Department of Epidemiology and Biostatistics, School of Public Health, Peking University, Beijing, China

**Keywords:** polycystic ovary syndrome, type 2 diabetes, pleiotropy, conditional FDR, conjunctional FDR

## Abstract

Genome-wide association studies (GWAS) have identified several common variants associated with polycystic ovary syndrome (PCOS). However, the etiology behind PCOS remains incomplete. Available evidence suggests a potential genetic correlation between PCOS and type 2 diabetes (T2D). The publicly available data may provide an opportunity to enhance the understanding of the PCOS etiology. Here, we quantified the polygenic overlap between PCOS and T2D using summary statistics of PCOS and T2D and then identified the novel genetic variants associated with PCOS behind this phenotypic association. A bivariate causal mixture model (MiXeR model) found a moderate genetic overlap between PCOS and T2D (Dice coefficient = 44.1% and after adjusting for body mass index, 32.1%). The conditional/conjunctional false discovery rate method identified 11 potential risk variants of PCOS conditional on associations with T2D, 9 of which were novel and 6 of which were jointly associated with two phenotypes. The functional annotation of these genetic variants supports a significant role for genes involved in lipid metabolism, immune response, and the insulin signaling pathway. An expression quantitative trait locus functionality analysis successfully repeated that 5 loci were significantly associated with the expression of candidate genes in many tissues, including the whole blood, subcutaneous adipose, adrenal gland, and cerebellum. We found that *SCN2A* gene is co-localized with PCOS in subcutaneous adipose using GWAS-eQTL co-localization analyses. A total of 11 candidate genes were differentially expressed in multiple tissues of the PCOS samples. These findings provide a new understanding of the shared genetic architecture between PCOS and T2D and the underlying molecular genetic mechanism of PCOS.

## 1 Introduction

Polycystic ovary syndrome (PCOS) is the most common endocrine disorder in women of reproductive age, with an adulthood prevalence estimated at 5.5–19.9% (Saei Ghare Naz et al., 2019). Extensive research suggests that hyperandrogenism and hyperinsulinemia, the two most important etiologies of PCOS, are related to dermatological abnormalities, metabolic dysfunction, and further irreversible clinical deterioration (Azziz et al., 2016). However, the main drivers of PCOS and biological mechanisms behind its etiology are not well-defined, posing unignorable challenges that continue to present for a clear diagnosis and precise management. Twin studies have demonstrated that PCOS is a heritable disease with heritability estimates of 38–71%, highlighting the polygenic genetic pattern (Vink et al., 2006). In a family study involving 29,736 daughters (age range 13–41 years), the female offsprings of mothers with PCOS had a 5-fold increased risk of developing PCOS (Risal et al., 2019). Moreover, utilizing human genotyping arrays, genome-wide association studies (GWAS) involve identifying associations of genotypes with phenotype individuals, leading to a better understanding of the genetic architecture of PCOS. However, the known genetic loci identified by current GWAS account for about 10% of the observed heritability of PCOS, which may, in part, be due to the limitations to GWAS, such as population stratification and extreme polygenicity of many traits (Stener-Victorin and Deng, 2021; Uffelmann et al., 2021). More risk loci with small effect sizes on PCOS remain to be discovered.

Leveraging the phenotypic and molecular information of co-morbid PCOS and type 2 diabetes (T2D) may improve the discovery of novel PCOS susceptibility loci. PCOS is the common co-morbidity experienced by adult women with T2D, with its prevalence over 30.0% (Conn et al., 2000; Kelestimur et al., 2006). Prospective studies have indicated that PCOS patients younger than 40 years have a 4–10 fold increase in risk for T2D (Kazemi Jaliseh et al., 2017; Liao et al., 2022). On the other hand, some research studies available have observed that insulin resistance and hyperinsulinemia involved in T2D can lead to overstimulation of the recruitment and growth of preantral and small antral follicles and the increasing risk of ovarian dysfunction, which contributes to PCOS, indicating an underlying pathophysiology association between PCOS and T2D (Thong et al., 2020). In addition, the significant clustering of T2D in the parents and siblings of PCOS probands from a family suggests a pivotal role of shared genetic factors in the two diseases (Yilmaz et al., 2018). However, evidence of the genetic association between PCOS and T2D is conflicting. The linkage disequilibrium (LD) score analysis showed a significant positive genetic correlation between PCOS and T2D ( $r_{g}=0.31$ ), whereas a Mendelian randomization study demonstrates that PCOS has a negative impact on T2D (OR = 0.88) (Day et al., 2018; Zhu et al., 2021). Significantly, the genetic correlation between PCOS and T2D, reported in previous studies, might not reflect the true pleiotropic action of genes on two phenotypes because it fails to capture the mixed directions of the effect across shared genetic loci (Frei et al., 2019). Pleiotropy between two traits, also called shared genetic architecture or genetic overlap, is a less stringent condition than genetic correlation, and it represents many variants that affect both traits simultaneously, regardless of their allelic effect directions (Bulik-Sullivan et al., 2015). However, no genetic study has explored the shared genetic architecture between PCOS and T2D.

In this study, to determine the shared polygenic architecture between PCOS and T2D with or without the adjustment of body mass index (BMI), we employed a bivariate causal mixture model (MiXeR) based on GWAS summary statistics of T2D and PCOS. Next, we applied the conditional/conjunctional false discovery rate (condFDR/conjFDR) method to identify novel genetic loci associated with PCOS. To explain the genetic variants associated with a gene expression phenotype, we assessed the expression quantitative trait locus (eQTL) functionality of the discovered loci. Finally, we performed a co-localization analysis with eQTL data and a differential gene expression analysis to detect the target genes in different tissues for a set of identified single nucleotide polymorphisms (SNPs).

## 2 Materials and methods

### 2.1 Participants

The GWAS summary statistics came from the most recently published large-scale GWAS meta-analysis for T2D and PCOS(Day et al., 2018; Mahajan et al., 2018). Data on PCOS were collected from seven cohorts of European descent. The PCOS sample consisted of 5,209 cases and 32,055 controls, excluding the self-report sample from the 23andMe database (*n* = 87,943) due to data availability. All PCOS cases were diagnosed based on the National Institute of Health (NIH) criteria or the Rotterdam criteria (Zawadzki et al., 1992; ESHRE and Group, 2004). After the quality control procedures for each study, genotypic data for the remaining SNPs were used by researchers to perform association analyses. Estimates of genetic variants across these studies were combined *via* a fixed-effect inverse-weighted–variance meta-analysis which was performed adjusting for age. Summary statistics on T2D, with and without adjustment for BMI, were obtained from the Diabetes Genetics Replication and Meta-Analysis (DIAGRAM) consortium. GWAS data from 32 cohorts comprised 74,124 cases and 824,006 controls of European ancestry. The case status was defined by an inclusive T2D diagnosis (e.g., diagnostic fasting glucose or HbA1c levels, hospital discharge diagnosis). With each study, all variants were tested for association with T2D in a regression framework under an additive model of the effects of the risk allele, and the results were merged using fixed-effects meta-analysis with the inverse-variance weighting of log ORs. More details can be found in the supplementary material.

### 2.2 Statistical analysis

#### 2.2.1 Quantification of the polygenic overlap between PCOS and T2D

To quantify the polygenic overlap between PCOS and T2D, we performed a MiXeR analysis using the GWAS data on both traits (Frei et al., 2019). First, we applied a univariate causal mixture model to generate two key parameters using the effect sizes, $\beta_{i}$ , for SNPs in PCOS and T2D (with and without adjustment of BMI) summary statistics: polygenicity (the proportion of non-null variants, π ) and discoverability (the phenotypic variance of non-null variant effect sizes, $\sigma_{\beta }^{2}$ ) (Frei et al., 2019). Second, three scenarios for the association of each SNP and both of the traits were assumed: 1) SNP affects both traits (shared SNP); 2) SNP affects only one of two traits (trait-specific SNP); 3) SNP has no effect on either trait (null SNP). Third, a bivariate causal mixture model was built under the assumption that all non-null variants followed the concordant distributions of the effect size to calculate the estimated number of shared and trait-specific causal variants, which explains 90% of SNP heritability in each trait. Dice coefficients for each pair of traits, an estimated percentage of the number of shared SNPs against all non-null SNP for both traits, were computed. We also assessed genome-wide genetic correlation ( $r_{g}$ ) across all SNPs between PCOS and T2D using the MiXeR model. Finally, to serve as a complement to $r_{g}$ estimation, cross-trait linkage-disequilibrium score regression (LDSC) for each pair of traits was employed under the hypothesis that the directions of the effect size of shared SNP are consistently aligned (Bulik-Sullivan et al., 2015). Additional information about the MiXeR model and the LDSC model can be found in the supplementary material.

#### 2.2.2 Data quality control and pre-processing

Prior to cond/conj FDR calculation, the data quality control and pre-processing procedures were implemented, as recommended by the authors who developed cond/conj FDR software Pleiofdr (Andreassen et al., 2013). We excluded the SNPs in the human major histocompatibility complex (MHC) region (hg19 as chr6: 25,119,106–33,854,733) and the 8p23.1 region (hg19 as chr8: 7,200,000–12,500,000) due to the characteristic high LD of SNPs within MHC regions and the large inversion polymorphisms harboring in the 8p23.1 region which is one of the characteristics in this extended block of LD (Antonacci et al., 2009; Bosch et al., 2009; Trowsdale and Knight, 2013). Including SNPs from two genetic regions may lead to bias in the subsequent analysis. We also applied a genomic control procedure to correct all *p*-values by the genomic inflation factor $\lambda_{GC}$ because it can minimize the impact of global variance inflation due to polygenic effects and provide a robust estimate of the null effects (Schork et al., 2013).

#### 2.2.3 Visualization of genetic pleiotropy enrichment

Using the summary statistics from PCOS and T2D GWAS without adjustment of BMI, we constructed quantile–quantile (Q–Q) plots for one phenotype based on varying levels of association with another phenotype under the null hypothesis to intuitively assess for pleiotropic enrichment of SNP association (Smeland et al., 2020). Specifically, we estimated the empirical cumulative distribution of nominal *p*-values obtained from GWAS summary statistics of one trait for all SNPs and the subsets of SNPs determined by significance levels below the indicated threshold for another trait ( $-log_{10}P>0$ , $-log_{10}P>1$ , $-log_{10}P>2$ , and $-log_{10}P>3$ corresponding to P<1, P<0.1, P<0.01, andP<0.001 , respectively). Q–Q plots of SNPs with nominal $-log_{10}P<7.3$ (corresponding to $P\;>\;5\;\times 10^{-8}$ ) were focused on assessing polygenic effects below the standard GWAS significance threshold. Pleiotropic enrichment exists if the Q–Q curve was plotted as successive leftward deflections from the null distribution, corresponding to a larger proportion of SNPs with a nominal $-log_{10}P$ value greater than or equal to a given threshold. To mitigate spurious enrichment resulting mainly from the LD structure across the human genome, we constructed all conditional Q–Q plots after random pruning averaged over 100 iterations. Random SNP in every LD block (defined by $r^{2}>0.1$ ) was selected, and the empirical cumulative distribution function was computed using the corresponding *p*-values at each iteration.

#### 2.2.4 Identification of PCOS-associated loci

To improve the detectability of genetic variants with smaller effect sizes that modulate the PCOS risk, we applied a condFDR statistical method using the GWAS summary statistics of PCOS and T2D without the adjustment of BMI (Smeland et al., 2020). As an extension of the standard FDR framework, the condFDR method integrated genetic association test statistics of one phenotype with another. Specifically, SNPs from the GWAS data on the primary phenotype were stratified based on different *p* values of the conditional trait. The posterior probability that a given SNP is null (has no association), given that its *p*-values for that SNP are less than or equal to the observed ones, was calculated. Next, we evaluated the per-SNP condFDR values of the PCOS conditioned on T2D. To further discover the SNP that is associated with PCOS and T2D simultaneously, the conjFDR procedure was further applied. The conjFDR framework is based on condFDR and is determined by the maximum condFDR values for PCOS, given T2D and vice versa. It calculates the posterior probability that a random SNP is null for either trait or both simultaneously, given that the observed *p*-values for both traits are less than or equal to the given *p*-values for each trait.

In order to include more candidate loci for further analysis, the thresholds of condFDR and conjFDR were both set to 0.05. Manhattan plots were also constructed based on ranking condFDR and conjFDR values to position the PCOS risk loci and the shared genetic risk loci. condFDR and conjFDR analyses were performed after random pruning for all SNPs across 100 iterations by selecting one random SNP per linkage disequilibrium block (defined by $r^{2}>0.1$ ). For more details, see the supplementary material.

#### 2.2.5 Functional annotation of PCOS-associated loci

We further used the SNP2GENE function of functional mapping and annotation (FUMA) protocol version 1.3, to define the lead SNPs and SNPs having a comparatively high LD ( $r^{2}\geq 0.6$ ) with corresponding lead SNP based on positional and chromatin interaction information of SNPs from 18 biological data repositories and tools (http://fuma.ctglab.nl/) (Watanabe et al., 2017). SNPs having a condFDR or conjFDR <0.05 and independent of each other at LD $\;r^{2}\;<0.6\;$ were identified as significant independent SNPs. Those SNPs independent of each other at LD $r^{2}\;<0.1$ were then selected as lead SNPs (or pleiotropic SNPs). The border for a genomic locus was defined as a region containing all candidate SNPs in LD ( $r^{2}\;\geq 0.6$ ) with at least a lead SNP. Candidate SNPs were merged into a genomic locus if the distances between them were less than 250 Kb. In addition, a novel risk variant associated with PCOS was defined as the lead SNP that 1) condFDR or conjFDR is less than 0.05; 2) *p*-value is greater than $\;5\;\times 10^{-8}$ in original PCOS GWAS and other PCOS GWAS research; and 3) independent of reported SNPs at LD $r^{2}\;<0.6$ and separated with reported SNPs by at least 250 Kb.

Gene mapping: two methods were performed to map SNPs to genes. First, positional mapping was performed using the SNP2GENE function of FUMA. Candidate SNPs in each genomic risk locus were assigned to their nearest genes based on functional annotations, namely, the combined annotation dependent depletion (CADD) score, probability of regulatory functionality (RegulomeDB score), and transcription/regulatory effects from chromatin states (the minimum chromatin state) (Boyle et al., 2012; Kircher et al., 2014; Roadmap Epigenomics Consortium et al., 2015). The CADD framework scores the deleteriousness of candidate SNPs by integrating 63 functional annotations by training a support vector machine, and the CADD score of an SNP greater than 12.37 indicates that the SNP is potentially deleterious (Kircher et al., 2014). RegulomeDB serves to predict whether candidate SNPs affect transcription factor binding and gene expression, and each SNP was assigned a rank score ranging from 1 to 7 (Boyle et al., 2012). A lower score for a candidate SNP represents stronger evidence of regulatory function. SNPs with RegulomeDB score ≤2 were defined as SNPs being functional. The minimum chromatin state was generated by using a multivariate hidden Markov model with 15 categorical states on the basis of five histone modification marks for 127 epigenomes to predict the accessibility of chromatin regions (every 200bp bin) (Roadmap Epigenomics Consortium et al., 2015). A lower score indicates higher accessibility of chromatin regions. Scores 1–7 refer to open chromatin states, representing that the genomic region where candidate SNPs are located is the open chromatin region which reflects the DNA regulatory potential of a genomic region. Second, we also utilized a variant-to-gene (V2G) tool developed by Mountjoy et al. (2021) to perform a mapping of lead SNPs (https://genetics.opentargets.org/). Specifically, the information from molecular phenotype quantitative trait locus experiments, chromatin interaction experiments, *in silico* functional predictions, and the distance between the variant and each gene’s canonical transcription start site was combined and then aggregated by taking the mean weighted-quantile to give an overall V2G score for each SNP–gene pair. The gene with the highest V2G score in a list of genes associated with a given SNP was considered as the mapped gene of it.

#### 2.2.6 Validation of expression quantitative trait locus functionality

eQTL analyses can help identify the associations between genetic variants and their corresponding gene expressions. It also facilitates the isolation of causal genes affecting PCOS. Therefore, we assessed the eQTL functionality of the identified PCOS loci in 49 tissues using publicly available data from the Genotype–Tissue Expression database version 8 (GTEx Release V8) (Lonsdale et al., 2013; Aguet et al., 2020). The fastQTL method was used to generate the candidate gene set associated with PCOS loci. The FDR threshold of less than 0.05 was applied to identify all significant cis-eQTLs, which were generally classified as variants within 1 Mb pairs of the gene transcription start site of the interested gene. Furthermore, a cis-eQTL analysis for identified PCOS loci was repeated using the whole-blood eQTL data from BIOSQTL and eQTLGen consortiums (Zhernakova et al., 2017; Võsa et al., 2021).

#### 2.2.7 Co-localization of GWAS and eQTL data

To advance the identification and prioritization of causal genes for PCOS, genetic co-localization analyses were conducted using the R package COLOC, based on combining PCOS GWAS meta-analysis data with eQTL data (https://cran.r-project.org/web/packages/coloc/). COLOC is a Bayesian-based method that produces the posterior probabilities of all possible configurations between two traits by performing an approximate Bayes factor computation (Giambartolomei et al., 2014). SNP associations for two traits were used to generate the posterior probability of five mutually exclusive hypotheses at a specific locus: 1) H0: neither trait has a causal SNP in the region; 2) H1: only the first trait, disease, has a causal SNP in the region; 3) H2: only the second trait, gene expression, has a causal SNP in the region; 4) H3: both traits are associated with different causal SNPs in the region; and 5) H4: two traits share a causal SNP in the region. For each co-localization, we extracted genetic variants available in both eQTL summary statistics of a testing gene and within 500 Kb of pleiotropic loci (250 Kb on each side of the pleiotropic SNPs) on four types of human tissues: whole blood, ovary, subcutaneous adipose, and visceral adipose (omentum) tissues. These single-tissue eQTL summary statistics were obtained from the GTEx and eQTLgen consortium (Aguet et al., 2020; Võsa et al., 2021). In the present study, we tested the nearest/mapped genes of pleiotropic SNPs and the genes (eGenes) whose significant eQTLs were overlapping with at least one of the pleiotropic SNPs. We used default prior (prior probabilities = $1\times 10^{-4}$ ) to PCOS associations ( $p_{1}$ ) and eQTLs ( $p_{2}$ ). As for the prior that a random SNP is associated with either GWAS or eQTL $\left(p_{12}\right)$ , we selected $1\times 10^{-5}$ as prior probabilities. However, in sensitivity analyses, $p_{12}$ of $5\times 10^{-6}$ was chosen to repeat the analysis, as recently proposed (Wallace, 2020). A posterior probability of ≥80% was considered sufficient to support one of the hypotheses.

#### 2.2.8 Differential gene expression analysis for PCOS-associated loci

Using the publicly available gene expression data provided by the Gene Expression Omnibus (GEO) database, we examined whether the nearest/mapped genes and eGenes of pleiotropic SNPs were differentially expressed in PCOS cases. Differential gene expression analyses of the four datasets, including GSE10946 (cumulus cells) (Kenigsberg et al., 2009), GSE98595 (granulosa cells) (Ferrero et al., 2018), GSE8157 (skeletal muscle) (Skov et al., 2008), and GSE48301(proliferative phase endometrium) (Piltonen et al., 2013), were performed using LIMMA. LIMMA is an R package for performing multiple linear regression models using microarray data (Smyth, 2004). The genes with *p*-values lower than 0.05 were considered as nominally differential expression genes. Further details about sample selection and statistical analysis can be found in the supplementary material.

**FIGURE 1:**
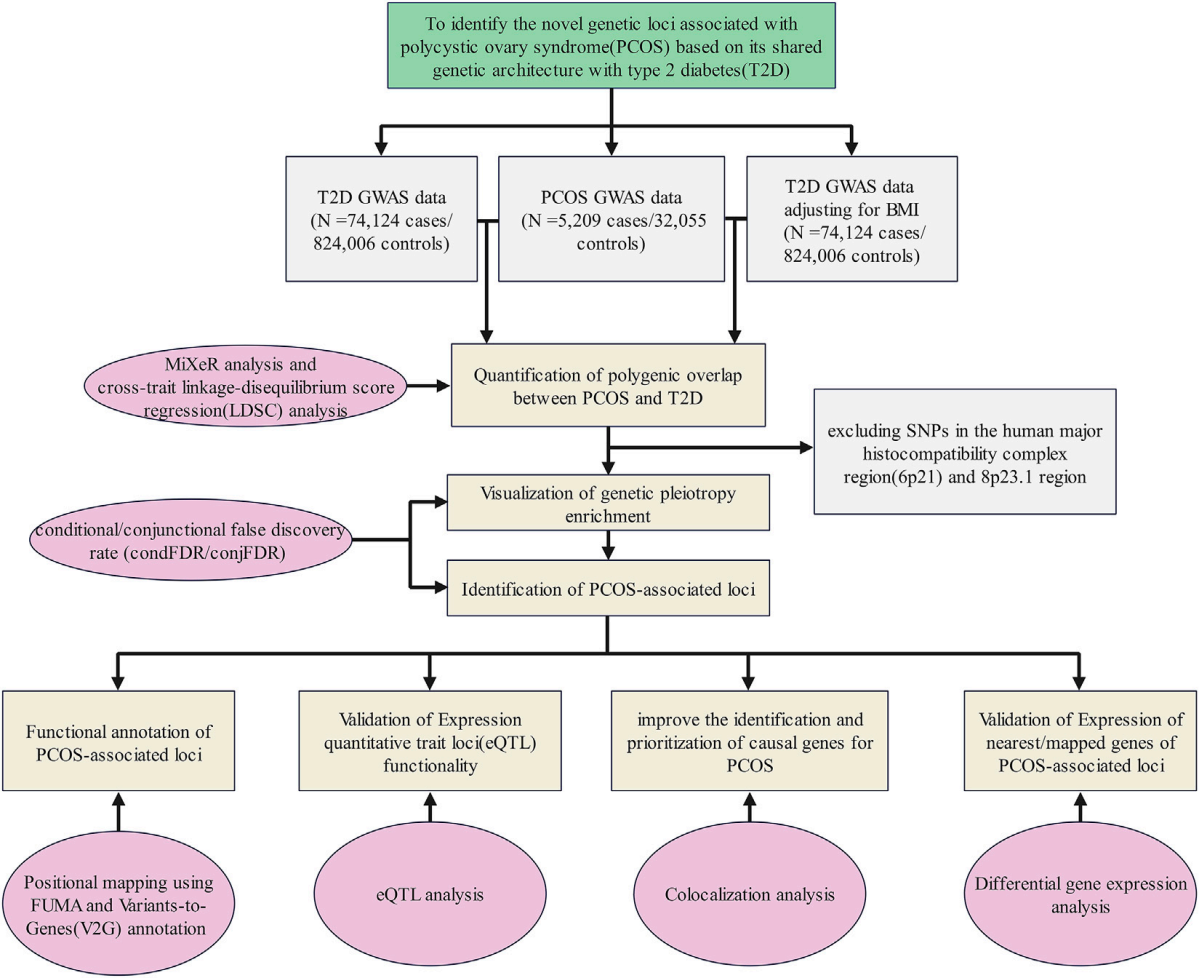
Flow diagram of the overall study design. GWAS summary statistics on PCOS and T2D with or without adjustment of BMI were collected from publicly available databases and open literature reports. We quantified the polygenic overlap between PCOS and T2D. Then, we visualized the genetic pleiotropy enrichment between both traits and identified the genetic loci associated with PCOS or/and T2D. Finally, four bioinformatics approaches were applied to explore the potential function of the identified genetic loci.

A flow diagram presents an overview of the study design (Figure 1).

## 3 Results

### 3.1 Polygenic overlap between PCOS and T2D

**FIGURE 2:**
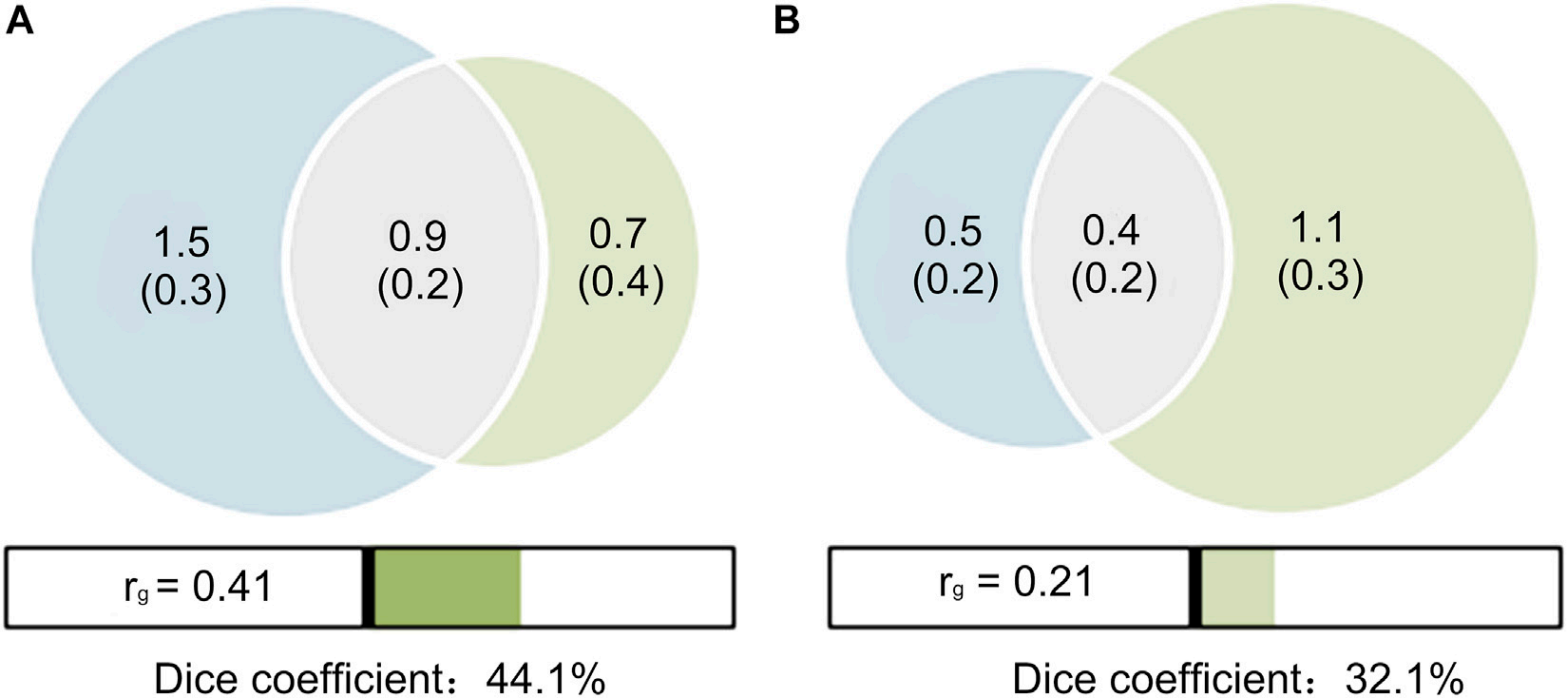
Venn diagrams of shared causal variants (gray). **(A)** T2D unadjusted by BMI (red) and PCOS (blue); **(B)** T2D adjusted by BMI (red) and PCOS (blue). The estimated numbers of causal variants are shown in thousands, explaining 90% of SNP heritability in each phenotype, followed by the standard error. The Dice coefficients, an overall measure on a 0–100% scale of the polygenic overlap, are 44.1% **(A)** and 32.1% **(B)**, respectively. The genetic correlations ( $r_{g}$ ) estimated by the MiXeR model are 0.41 **(A)** and 0.21 **(B)**, respectively.

A MiXeR analysis was performed between PCOS and T2D with and without adjustment for BMI. The results were shown as Venn diagrams, providing preliminary evidence of the polygenic overlap between PCOS and T2D (Figure 2). In the unadjusted scenario (Figure 2A), of the 1.6 K causal variants linked to PCOS, 0.9 K ( standard error(se)=0.2 ) are shared with T2D (3.1K, overall). The overall measure of the polygenic overlap, quantified by the dice coefficient on a 0–100% scale, is 44.1%. The genome-wide level genetic correlation ( $r_{g}$ ) between PCOS and T2D is 0.41, according to MiXeR, or 0.31 according to cross-trait LDSC (Supplementary Table S1). In the scenario of adjusting for BMI in original T2D GWAS data (Figure 2B), MiXeR estimated a much lower number of causal SNPs, but it still indicates a moderate polygenic overlap between PCOS and T2D. Of the 1.5 K causal variants of PCOS, 0.4 K ( se=0.2 ) are shared with T2D (2.0K, overall), with a dice coefficient of 32.1%. We also found a positive genetic correlation between PCOS and T2D: ( $r_{g}=0.21$ according to MiXeR, or $\;r_{g}=0.12$ according to cross-trait LDSC $\;r_{g}=0.12$ (Supplementary Table S1)). In addition, the positive AIC (Akaike information criterion) value, a model selection criterion that evaluates the quality of the MiXeR model compared with each of the other models, indicates that the summary statistics of PCOS and T2D had enough statistical power to fit the MiXeR model (Supplementary Table S1).

### 3.2 Enrichment of PCOS conditional on T2D and vice versa

**FIGURE 3:**
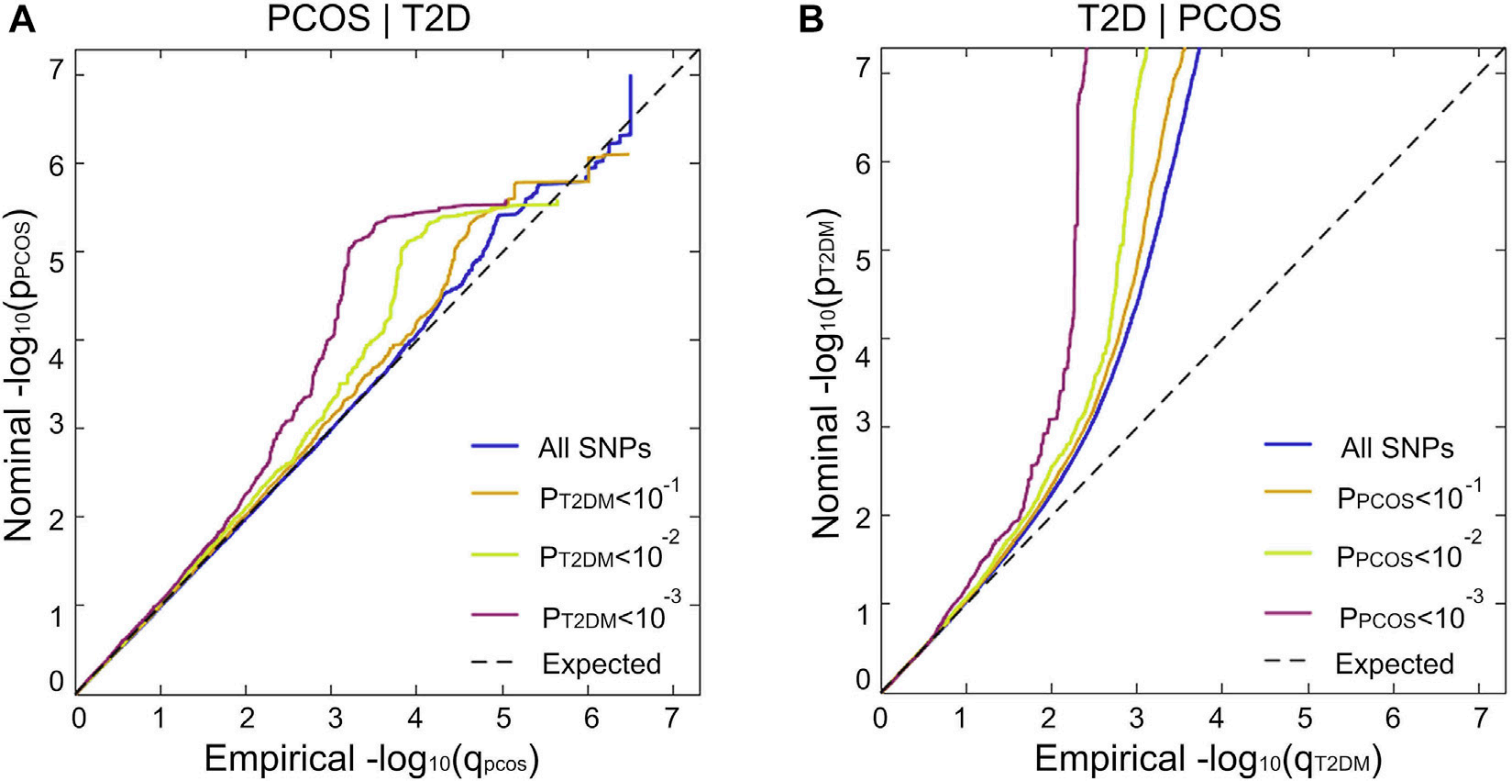
Genetic pleiotropy enrichment of PCOS conditional on T2D (without adjustment for BMI) and vice versa. Stratified conditional Q–Q plot of nominal versus empirical and negative log10-transformed *p*-values in the primary phenotype as a functional significance of association with the secondary phenotype at the level of *p* < 1(All SNPs, blue), *p* < 0.1 (orange), *p* < 0.01 (green), and *p* < 0.001 (purple) corresponding to $-log_{10}P>0,\;-log_{10}P>1,\;-log_{10}P>2,and\;-log_{10}P>3$ , respectively. **(A)** PCOS conditioned on T2D; **(B)** T2D conditioned on PCOS. Dotted lines demonstrate the null hypothesis. The summary statistics of T2D without adjustment of BMI were selected for plotting those conditional Q–Q plots.

The stratified conditional Q-Q plot shows a successive increment of SNP enrichment for PCOS conditioned on association *p*-values for T2D and vice versa (Figure 3). Successive leftward shifts for the strata of SNPs with higher significance in T2D indicate that the proportion of PCOS-associated SNPs increases considerably with higher levels of association with T2D, suggesting an underlying shared genetic architecture between PCOS and T2D (Figure 3A). The reverse stratified and conditional Q–Q plots also display genetic enrichment for T2D, given PCOS (Figure 3B).

### 3.3 PCOS-associated loci identified by the condFDR and conjFDR methods

**FIGURE 4:**
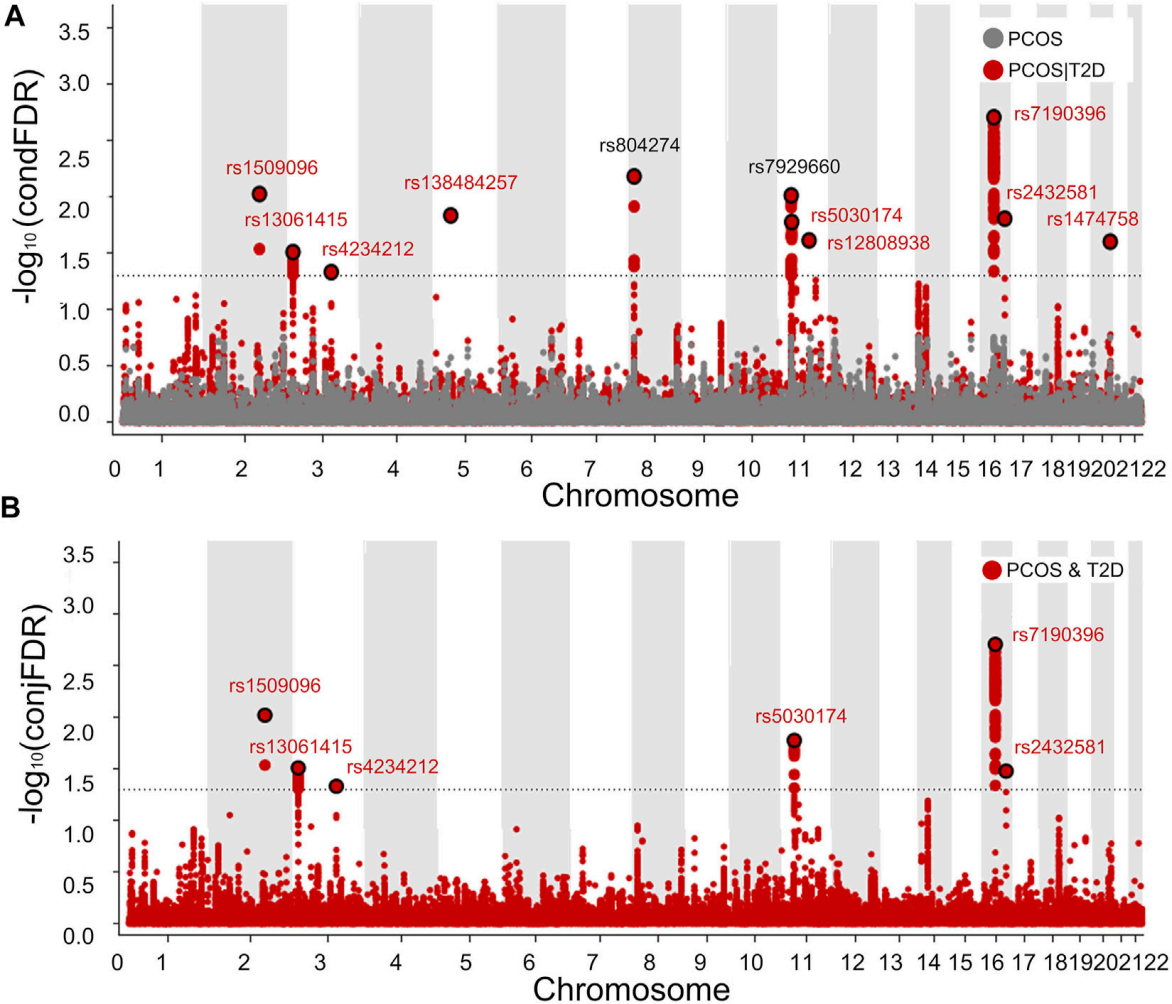
Manhattan plot showing 11 genetic loci associated with PCOS or/and T2D. Manhattan plots showing the negative log10-transformed condFDR values **(A)** and negative log10-transformed conjFDR values **(B)** for each SNP on the *y* axis and chromosomal positions along the *x* axis. The most significant SNP lead in each LD block is enlarged and encircled in black, whereas the small points represent other SNPs. Labels correspond to lead SNPs previously reported for published loci (black) and for newly identified loci (red).

Based on the polygenic overlap between PCOS and T2D, we identified specific SNPs related to PCOS by combining the information on SNP associations available in PCOS and T2D GWAS summary data. The results of condFDR and conjFDR were visualized in two Manhattan plots, in which all SNPs without pruning are shown (Figure 4). Using condFDR<0.05 and after pruning the SNPs for LD at $r^{2}\;>\;0.1$ , we identified 11 loci associated with PCOS conditioned on T2D (Table 1; Figure 4A). At conjFDR <0.05 and after pruning the SNPs, 6 of the 11 loci detected by condFDR are associated with both PCOS and T2D (Figure 4B). By comparing the directions of the allelic effects, as denoted by the sign of the z-scores, of lead SNP at detected PCOS-associated loci, we discovered that 6 lead SNPs (namely, rs1509096, rs13061415, rs12808938, rs7190396, rs2432581, and rs1474758) have consistent effect directions in PCOS and T2D, and 5 lead SNPs (namely, rs4234212, rs138484257, rs804274, rs7929660, and rs5030174) have opposite effect directions (Table 1). Of the 11 PCOS-associated loci, 9 were novel whose lead SNPs contain rs1509096, rs13061415, rs12808938, rs7190396, rs2432581, rs1474758, rs4234212, rs138484257, and rs5030174 (Table 1).

**TABLE 1:** Genetic susceptibility loci associated with PCOS identified by the conditional FDR and conjunctional FDR methods.

Loc^a^	CHRPOS^b^	SNP	Ref/Alt^c^	Functional category	Nearest gene	Mapped gene^d^	${\text{FDR}}_{\text{PCOS}|\text{T}2\text{D}}$ ^g^	${\text{FDR}}_{\text{PCOS}\&\text{T}2\text{D}}$ ^h^	GWAS P-value^f^		GWAS Z-score^i^	
									T2D	PCOS	T2D	PCOS
1	2:165,737,889	rs1509096	G/A	intergenic	RNA5SP111	SLC38A11	9.43E-03	9.53E-03	1.80E-05	1.40E-05	−4.34	−4.29
2	3:12,349,924	rs13061415	C/T	intronic	PPARG	PPARG	3.09E-02	3.09E-02	6.60E-09	5.60E-05	4.03	5.80
3	3:123,010,775	rs4234212	C/T	intronic	ADCY5	SEC22A	4.64E-02	4.64E-02	5.80E-05	9.30E-05	3.91	−4.02
4	5:43,618,391	rs138484257	G/T	intronic	NNT	NNT	1.46E-02	9.69E-01	5.30E-01	9.80E-08	−5.33	0.63
5	8:11,625,205	rs804274	C/A	intergenic	NEIL2	CTSB	6.60E-03	7.47E-01	7.30E-02	2.10E-07	−5.19	1.79
6	11:30,339,461	rs7929660	G/A	intergenic	ARL14EP	ARL14EP	9.72E-03	5.65E-01	2.70E-02	6.80E-07	−4.97	2.21
7	11:32,449,098	rs5030174	G/A	intronic	WT1	WT1	1.67E-02	1.67E-02	1.50E-05	2.70E-05	−4.20	4.33
8	11:83,562,895	rs12808938	G/T	intronic	DLG2	DLG2	2.43E-02	6.11E-01	3.50E-02	1.60E-06	4.80	2.11
9	16:53,822,502	rs7190396	G/T	intronic	FTO	FTO	1.97E-03	1.97E-03	7.60E-74	2.50E-06	−4.71	−18.18
10	16:81,463,967	rs2432581	G/A	intergenic	CMIP	CMIP	1.56E-02	3.32E-02	1.00E-04	2.50E-05	4.21	3.89
11	20:56,125,891	rs1474758	C/A	intergenic	PCK1	PCK1	2.49E-02	9.17E-01	3.50E-01	2.50E-07	−5.16	−0.93

Note:

a Loc, locus.

b CHRPOS, chromosome position in the human reference genome build37 (or hg19).

c Ref/Alt, reference allele/alternative allele.

d Mapped gene, gene that is most likely to be associated with lead SNP, defined by the V2G score.

e FDR_PCOS|T2D_, conditional FDR of PCOS conditioned on T2D.

f FDR_PCOS&T2D_, conjunctional FDR of PCOS and T2D.

### 3.4 Gene definition and functional annotation

**FIGURE 5:**
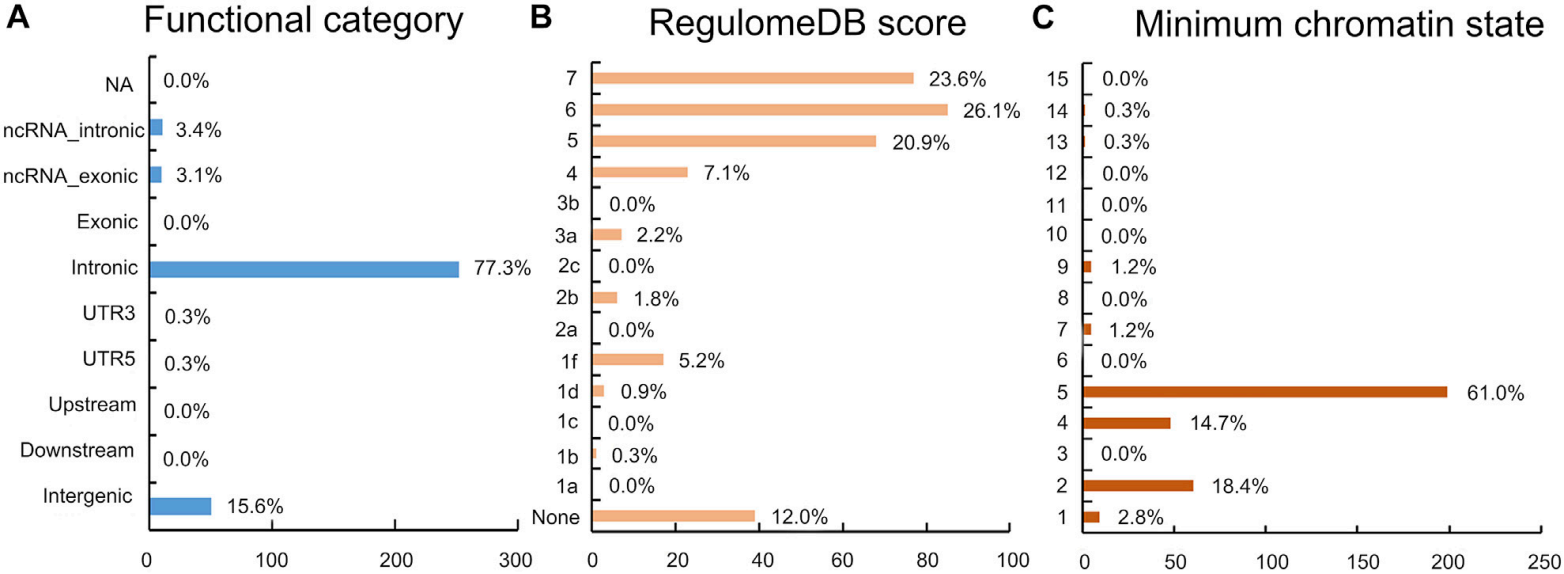
Distribution of the functional annotation for candidate SNPs. **(A)** Distribution of the functional consequences of independent SNPs. **(B)** Distribution of RegulomeDB scores, with a lower score indicating a higher likelihood of having a regulatory function. **(C)** Minimum chromatin state across 127 tissue and cell types for SNPs in a shared genomic region, with lower states indicating higher accessibility and states 1–7 referring to open chromatin states.

The functional annotation of all candidate SNPs in the 11 pleiotropic loci (*n* = 405; Figure 5) demonstrates that the majority are mostly intronic (64.8%) or intergenic (26.3%), while no SNP was found in any exons (Figure 5A and Supplementary Table S2). For the 11 top lead SNPs in the pleiotropic loci associated with PCOS conditioned on T2D, we mapped the nearest gene to them based on positional information and functional annotation and found that six lead SNPs are located inside a protein-coding gene and five lead SNPs between the genes (nearest gene in Table 1). Specifically, rs13061415, rs4234212, rs138484257, rs5030174, rs12808938, and rs7190396 are located within the *PPARG (OMIM 601487), ADCY5(OMIM 600293), NNT (OMIM 607878), WT1(OMIM 607102), DLG2(OMIM 603583), and FTO (OMIM 610966)*, respectively. On the other hand, rs1509096 (nearest gene: RNA5SP111; HGNC 42909), rs804274 (nearest gene: NEIL2; OMIM 608933), rs7929660 (nearest gene: ARL14EP; OMIM 612295), rs2432581 (nearest gene: CMIP; OMIM 610112), and rs1474758 (nearest gene: PCK1; OMIM 614168) occur between genes (Table1 and Supplementary Table S2).

Considering that the positional mapping of pleiotropic loci using FUMA might not be the best strategy to select the potential causal genes of diseases, the V2G tool was also applied to identify the most probable causal genes having a biological effect on PCOS. By integrating multiple sources of information produced by different experimental methods, eight pleiotropic loci are assigned to eight candidate genes identical to the results generated from the positional mapping method (mapped gene in Table 1). However, rs1509096, rs4234212, and rs804274 were assigned to *SLC38A11* (OMIM 616526), SEC22A (OMIM 612442), and CTSB (OMIM 116810) using the V2G tool.

No lead SNP has a CADD score above 12.37. However, of the 405 candidate SNPs, 17 SNPs in strong LD ( $r^{2}\geq 0.8$ ) with four lead SNPs (rs13061415, rs5030174, rs7190396, and rs7929660) have CADD scores above the 12.37 threshold, suggestive of high deleteriousness (Supplementary Table S2). In addition, 7.4% of candidate SNPs (*n* = 30, RegulomeDB scores ≤ 2) are likely to affect binding (Figure 5B and Supplementary Table S2). A lead SNP (rs13061415) reports a RegulomeDB score of 1f, indicating that it possibly affects transcription factor binding. The distribution of the minimum chromatin state showed that 95.6% of candidate SNPs (*n* = 387) are located in open chromatin state regions (Figure 5C and Supplementary Table S2). In total, 8 lead SNPs(rs1509096, rs13061415, rs804274, rs7929660, rs12808938, rs7190396, rs2432581, rs1474758) scored 5 separately, representing that they may be involved in weak transcription of genes. rs4234212 and rs138484257 (score 4) are associated with a strong transcription and rs5030174 (score 2) to flanking active transcriptional start sites (TSS) (Supplementary Table S2).

### 3.5 eQTL functionality of pleiotropic SNPs

We investigated the gene regulatory effects of the 11 lead SNPs using the GTEx database. The results showed that eight lead SNPs are significantly associated with the expressions of multiple genes (eGenes) and were defined as eQTL (Supplementary Table S3). For example, rs1509096 is associated with the expressions of *SCN2A* and *SLC38A11* genes in the subcutaneous adipose, thyroid, and skeletal muscle tissue; rs13061415 is associated with the expressions of *PPARG*, *TIMP4*, and *HMGCS1* genes in the human brain and transverse colon tissues (Supplementary Table S3). Next, we replicated the investigation of eQTL functionality of lead SNPs using eQTLGen and BIOSQTL databases. Among the eGenes identified by the GTEx database, 8 of 15 genes were repeatedly reported, and their expressions were associated with five pleiotropic SNPs (rs1509096, rs4234212, rs13061415, rs7929660, and rs804274) in the whole blood tissue. In total, eQTL analyses identified 23 eGenes whose expressions were significantly associated with eight lead SNPs.

### 3.6 Co-localization of eQTLs at 11 pleiotropic loci

We examined the overlap between eQTLs and pleiotropic loci and performed a genetic colocalization analysis to explore whether pleiotropic loci co-localized with 27 candidate genes containing the nearest/mapped genes of pleiotropic SNPs and the eGenes. Of the 11 pleiotropic loci, one locus, whose lead SNP is rs1509096, has high support ( PP4 = 89.60% ) for co-localization with *SCN2A* gene in the subcutaneous adipose tissue (Supplementary Table S4). Additionally, in the genomic region of 9 pleiotropic loci, the causal variants of PCOS are inconsistent with the causal variants that regulate the gene expression ( PP3≥ 80.00% ) in the whole blood, ovary, and adipose tissues *(FDFT1, BLK, FAM167A, NEIL2, CTSB, FAM86B, RP11-297N6.4, ARL14EP, and FTO)*. After applying a more stringent prior ( $p_{12}=$ 5 $\times 10^{-6}$ ), those pleiotropic loci with high support for hypothesis 3 or hypothesis 4 ( PP3≥ 80% or PP4≥80% ) remained (Supplementary Table S4).

### 3.7 Differential expression of the candidate genes

After accessing the gene expression profile data from the GEO dataset, we extracted the mRNA expression values for 26 candidate genes except for *RP11-297N6.4* gene and performed differential gene expression analyses. In total, 11 candidate genes were identified as significantly differentially expressed between PCOS-associated tissues and normal tissues ( P<0.05 ) (Supplementary Table S5). Specifically, seven genes, namely, *CMIP*, *CTSB*, *C8orf49*, *DLG2*, *FTO*, *NNT*, and *TIMP4* are significantly downregulated in PCOS-associated tissues, while other genes were significantly upregulated in PCOS-associated tissues (Supplementary Table S5).

## 4 Discussion

In recent years, GWAS have identified some unique associations between SNPs and PCOS, but several SNPs with minor genetic effects remain to be identified. By leveraging information on the GWAS summary data from PCOS and T2D, we estimated the polygenic overlap between two phenotypes using MiXeR models and LDSC models. We observed a moderate polygenic overlap between PCOS and T2D, regardless of whether the SNP associations of T2D were adjusted by BMI. Stratified Q–Q plots further support the evidence for the polygenic overlap between PCOS and T2D. In the LDSC model, the estimate of the genetic correlation between PCOS and T2D is significant and positive, similar to the results from the MIXeR model, implying that genes that increase PCOS also increase the risk of diabetes. The cond/conjFDR framework is a powerful method to explore novel genetic susceptibility loci associated with PCOS by integrating two genetically correlated traits. We successfully detected 11 PCOS-associated loci conditional on T2D with a mixture of allelic effect directions. Of those, nine loci were novel, and six loci were jointly associated with PCOS and T2D. These findings strengthen prior genetic evidence (Hayes et al., 2015; Day et al., 2018). We discovered five cis-eQTLs near 15 candidate genes in multiple human tissues and successfully validated eight eQTL associations in other eQTL data. A co-localization analysis detected that 1 locus (rs1509096) has strong evidence for co-localization with the *SCN2A* gene in the subcutaneous adipose tissue. Furthermore, the differential gene expression analysis found that 11 of all the candidate genes were significantly differentially expressed in PCOS women compared with those of controls. These findings support the importance of abnormal gene expression in shared etiological mechanisms between PCOS and T2D.

For a pair of traits, a polygenic overlap refers to the fraction of genetic variants affecting both traits simultaneously over the total number of causal variants across the two traits observed regardless of their allelic effect directions. Few studies have estimated the degree of genetic overlap or the number of shared underlying causal variants between PCOS and T2D despite some metrics used to compute the genetic correlation. We reported a moderate polygenic overlap between PCOS and T2D. Using cross-trait LDSC, PCOS shows a moderate positive genetic correlation with T2D, in agreement with previous studies (Zhu et al., 2021). It should be noted that genetic correlation may be statistically significant only if plenty of shared causal variants for both traits reveal consistent directions of effect sizes (same or opposite) (Frei et al., 2019). These findings suggest that the bulk of the shared causal variants are positively associated with PCOS and T2D and provide a complete understanding of the shared genetic architecture between PCOS and T2D, spanning numerous susceptibility genes of the two phenotypes that remain unknown. However, after adjusting for BMI in the original T2D GWAS data, the estimated number of shared causal SNPs simultaneously associated with PCOS and T2D decreased by approximately half, and the percentage of shared causal SNPs (Dice coefficient) decreased by one-third according to the MIXeR analysis. Notably, PCOS GWAS data collected in this study are not adjusted for obesity-related traits (BMI, etc.) because of data availability. If adjusted for BMI, these parameters may further decrease. These results reveal that a portion of the shared genetic architecture between PCOS and T2D may be associated with obesity. A study by Liu et al. (2022) has supported our results: rs2432581, identified as SNP shared between PCOS and T2D in the present study, was found to be a causal variant simultaneously associated with PCOS and WHR (waist-hip rate). The mapped genes (FTO, SLC38A11, and RNA5SP111) of the identified PCOS SNPs in our study were also associated with obesity-related traits, including WHR, WHR adjusting for BMI, and childhood BMI (CBMI), suggesting the shared genetic architecture of PCOS, T2D, and obesity (Liu et al., 2022). It partly explains why overweight or obese women with PCOS are more likely to experience T2D (Kakoly et al., 2019).

PCOS is a complex disease resulting from a complicated combination of genetic, epigenetic, and maternal-fetal environmental factors. Hyperandrogenism, which is the most prominent and heritable phenotypic trait, may be involved in the abnormal response to negative feedback regulation in the hypothalamic-pituitary-ovarian (HPO) axis and follicular follicle-stimulating hormone (FSH) resistance (Legro et al., 1998; Azziz et al., 2016). Hyperinsulinemia may impair the negative feedback regulation on the hypothalamic-pituitary-adrenal (HPA) axis and lead to further imbalance of HPO axis regulation by promoting an adrenal secretion of androgen (Wang et al., 2019b). However, questions remain as to the biological mechanisms underlying these symptoms. Two lead SNPs have not been reported in previous PCOS GWAS but were mapped to ARL14EP (rs7929660) and NEIL2/CTSB (rs804274), three candidate genes for PCOS(Hayes et al., 2015; Day et al., 2018; Tyrmi et al., 2022). rs7929660 is an eQTL for ARL14EP in 20 types of human tissues and is highly correlated with rs11031005 (LD $r^{2}=0.81$ ) and rs11031006 (LD $r^{2}=0.80$ ). The 11p14.1 locus harboring the ARL14EP gene has been related to endometriosis (Sapkota et al., 2017). rs11031005 and rs11031006 are known to be associated with reproduction-related phenotypes, including the length of the menstrual cycle and sex hormone levels [FSH](Ruth et al., 2016; Laisk et al., 2018). rs804274 is also an eQTL for *NEIL2* and *CTSB* genes. The *CTSB* gene encodes cathepsin B, a lysosomal cysteine protease. It has been reported that a high activity *CTSB* gene serves as proapoptotic in mouse ovarian cells, inhibiting the granular cell proliferation *via* inhibition of the p-Akt and p-ERK1/2 pathways (Chen et al., 2021). Mendelian randomization analysis has discovered that the *NEIL2* gene was potentially causally associated with PCOS (Sun et al., 2022). Statistical evidence, including the differential gene expression analysis we carried out in our research, suggests that *NEIL2* and *CTSB* could play an essential role in PCOS, although the underlying biological functions are still unclear.

In this study, the strongest novel signal of shared genetic effects between PCOS and T2D is rs7190396, located at FTO. FTO is an obesity susceptibility gene and encodes 2-oxoglutarate and Fe (Ⅱ)-dependent demethylase catalyzing the 3-methylthymine in single-stranded DNA and 3-methyluracil and 6-methyladenosine in RNA for repairing and modifying multiple nucleic acids (Loos and Yeo, 2014). rs7190396 is an eQTL for FTO in the skeletal muscle and was reported to be strongly associated with menarche in the United Kingdom Biobank GWAS ( $P\;=\;3\;\times 10^{-35}$ ), which might partly explain the late menarche phenotype in PCOS patients in a prospective cohort study (Bycroft et al., 2018; Tabassum et al., 2021). *FTO* gene plays an essential role during the evolution of many reproductive phenotypes, such as ovarian aging. An *in vitro* model showed that FTO knockdown could induce the faster aging process of granular cells by increasing the total amount of multifunctional N6-methyladenosine, indicating a key effect of FTO in abnormal ovulation processes (Jiang et al., 2021). However, the evidence that FTO is related to PCOS appears contradictory. A study showed that FTO upregulation could induce the dysfunction of ovarian granular cells by upregulating FLOT2 (Zhou et al., 2021). However, the functional mechanism of how rs7190396 or FTO affects PCOS is unknown, which can be explored in follow-up functional studies.

rs1509096 was assigned to RNA5SP111 or SLC38A11 according to two mapping methods. RNA5SP111 is a 5S ribosomal pseudogene (Cunningham et al., 2021), while SLC38A11 encodes solute carriers that transport amino acids as their primary substrate to engage in amino acid sensing and signaling in cells (Hellsten et al., 2018). In the present study, rs1509096 shows a strong eQTL effect on SLC38A11 and SCN2A. A transcriptomic study showed that a low expression of SLC38A11 could inhibit the regeneration of the endometrium cycle-to-cycle (Spitzer et al., 2012). Abnormal endometrial cell proliferation is more likely to be observed in PCOS women, probably resulting from the dysfunction of the HPO axis (Spitzer et al., 2012). In addition, we found that the *SCN2A* gene is co-localized with PCOS in subcutaneous adipose. A previous study showed that the expression of SCN2A might have positive effects on activating Na + channels in the human nervous system, but the relationship between SCN2A and PCOS is unclear (Sanders et al., 2018). The effect allele A of rs2432581 near CMIP (C-Maf-inducing protein, a negative regulator of T cell signaling) was positively associated with PCOS and T2D. CMIP could decrease the reactivity of T cells in response to CD3-CD28 stimulation and impede an appropriate T-cell activation in response to pathogens, which may explain the low-grade chronic inflammation in PCOS (Bannigida et al., 2020; Oniszczuk et al., 2020).

More importantly, rs13061415, a novel SNP located at PPARG and jointly correlated with T2D and PCOS, may affect transcription factor binding because of its low RegulomeDB score. PPARG (PPAG-γ) encodes a member of the ligand-dependent nuclear hormone receptor family of nuclear receptors, regulating adipogenesis through its interaction with several co-activators (Grygiel-Górniak, 2014). PPARG has been reported to be closely associated with PCOS in the European population (Zaki et al., 2017). Previous studies have showed that Pro12Ala and His447His polymorphisms of the PPARG might be protective factors of insulin resistance in PCOS women (Yilmaz et al., 2006; Shaikh et al., 2013). In fact, reduced fertility is more likely to be observed in mice with a specific deletion of PPARG in granular cells, which is a critical regulator of reproduction and development (Cui et al., 2002; Yang et al., 2008). DLG2, where rs12808938 is located, encodes a protein that forms a heterodimer with a related family member that may interact at postsynaptic sites for clustering of receptors, ion channels, and associated signaling proteins (Ali et al., 2018). There is limited evidence for the association between DLG2 and PCOS. However, a study on markedly delayed puberty reported that variants in DLG2 decrease the gonadotropin-releasing hormone expression of the hypothalamic cell line *in vitro* experiment, indicating that DLG2 may be associated with the regulation of the HPO axis (Jee et al., 2020).

In addition, we identified a novel pleiotropic SNP, rs1474758, which was mapped to the *PCK1* gene. In the liver and kidney, PCK1 encodes the gluconeogenic enzyme (PEPCK-C) that catalyzes the limiting-velocity step of the hepatic gluconeogenic pathway and functions in adipocytes of the glyceroneogenesis pathway (Beale et al., 2004). PCK1 might influence specific FSH-related processes, which could occur in PCOS. Some research studies indicated that PCK1 was involved in the fructose-1,6-bisphosphatase 1 signaling pathway and was highly expressed after the stimulation of a high level of FSH or testosterone (Perlman et al., 2006; Liu et al., 2017). The SNPs contributing to the risk of PCOS and T2D with opposite directions of effects cannot be ignored. We discovered three interesting novel loci with discordant directions of the effect for PCOS and T2D. The alleles T of rs138484257 and A of rs5030174 were associated with a decreased risk of T2D and an increased risk of PCOS. NNT, where rs138484257 is located at, encodes nicotinamide nucleotide transhydrogenase which produces high concentrations of NADPH for radical detoxification (Meimaridou et al., 2012). In animal experiments, NNT mutations could modulate the effect of Gclm gene deletion on the fertility of female mice (Nakamura et al., 2011). Although the relationship between NNT and PCOS is unknown, the NADPH pathway might play an essential role in granular cells. The results from a study suggest a harmful effect of overactive NADPH oxidase on the oocyte quality of PCOS women (Lai et al., 2018). WT1, where rs5030174 is located at, encodes Wilms’ tumor gene 1 protein that influences cellular development and cell survival as a transcription factor (Hamilton et al., 1995). A previous study has demonstrated that WT1 activation is necessary to reduce the premature apoptosis of granular cells in follicles *via* the activation of the β-catenin signal pathway (Wang et al., 2019a). The expression of WT1 was moderately correlated with testosterone, luteinizing hormone levels, and the antral follicle counts in a case control study (Wang et al., 2018). Last, the allele T of rs4234212, mapped to ADCY5 or SEC22A, was associated with an increased risk of T2D and a decreased risk of PCOS. rs4234212 is an eQTL for both ADCY5 and SEC22A. ADCY5 encodes membrane-bound adenylyl cyclase enzyme-5 that converts adenosine triphosphate to the cyclic adenosine monophosphate and pyrophosphate and regulates glucose-induced insulin secretion (Lin et al., 2020). Horikoshi et al. (2013) reported that variants in ADCY5 were associated with lower birth weight in the European population, implying that ADCY5 may be linked to a poor maternal–fetal environment. Furthermore, SEC22A, also known as a vesicle-trafficking protein SEC22 homolog B, is involved in vesicle trafficking and regulates multiple signaling and transportation pathways (Sun et al., 2020). In an animal model, SEC22A was found to be over-expressed in immature oocytes compared to matured counterparts, indicating a potential effect of SEC22A in oocyte growth and maturation (Mamo et al., 2011).

Our study has several strengths. It is worth noting that the prior pleiotropic approach (such as LD-score–based partitioned heritability) cannot capture the authentic shared genetic architecture if the shared SNPs with mixed effects exist in two traits. Therefore, we introduced the MiXeR tool. The advantage of this tool is that it extends the cross-trait LD score regression by incorporating a causal mixture model, capturing the mixture of the effect directions across shared genetic variants rather than measuring the overall genetic correlation (Frei et al., 2019). Second, to the best of our knowledge, this is the first study to report the results of cond/conj FDR, and we identified nine genetic variants associated with PCOS, which have never been reported in previous studies. Our results partly underpin the missing heritability of PCOS. Combining GWAS data from two traits using the cond/conjFDR approach increases the power to detect SNPs associated with common biological mechanisms and elucidates the shared pathophysiological relationships between the phenotypes. Furthermore, the application of the eQTL analysis, co-localization analysis, and differential gene expression analysis not only partly validates our results but also provides us with statistical evidence to verify the causal effect of pleiotropic loci on PCOS and T2D.

Several limitations need to be acknowledged. First, the self-report samples from the 23andMe database were removed, owing to data availability. Hence, the sample size of PCOS was comparatively small compared to T2D (PCOS *n* = 37,264 versus T2D *n* = 898,130), underpowering the MiXeR analysis and condFDR/conjFDR. More shared loci between PCOS and T2D are expected to be discovered when larger samples fulfilling the NIH or Rotterdam diagnostic criteria are available in PCOS GWAS. Second, PCOS consists of four phenotypes according to three clinical features: hyperandrogenism (either biochemical or clinical), ovulatory dysfunction, and polycystic ovarian morphology (Azziz et al., 2016). The results reported in our study represent the combined genetic effect of identified variants. A stratified analysis by the four subtypes mentioned previously cannot be performed, owing to data availability. Moreover, categorizing PCOS using clinical features may ignore the heterogeneity caused by different biological pathways. In the future, additional information such as different levels of omics information should be collected and combined to identify PCOS subtypes and determine their subtype-specific genetic variants. Third, the identified SNPs are probably not causal variants but tagged ones located in specific genomic regions, although the co-localization method was performed to detect potential causal SNPs for both traits. Fourth, all participants included in this study were of European ancestry, which did not reflect the trans-ancestry groups’ differential genetic backgrounds. Fifth, these loci identified by cond/conj FDR remain subjected to the same scrutiny as regular GWAS and sex-specific GWAS. Therefore, larger sample sizes are required in GWAS to achieve an adequate statistical power and determine the sex-specific genetic effect of identified loci, providing additional information on the pathophysiology of PCOS and its association with T2D. Finally, the underlying mechanism by which these loci play a role in PCOS development is still unclear. Functional mechanistic studies will be employed to determine the clinical significance of these loci in the future.

## 5 Conclusion

In conclusion, we reported a moderate polygenic overlap between T2D and PCOS, extending the current understanding of the common genetic variants influencing the two diseases. Present results also imply an essential role of BMI in two diseases. More importantly, we successfully improved the identification of pleiotropic genetic variants of PCOS and T2D, including nine novel loci. The results of the eQTL analysis, colocalization analysis, and differential gene expression analysis suggested that most loci are potential regions that regulate PCOS and T2D simultaneously. Our study may provide us with an improved understanding of the potential genetic mechanisms in PCOS.

## Data Availability

Summary-level T2D data are available at the DIAGRAM consortium website http://www.diagram-consortium.org/. Summary-level PCOS data are available at the Apollo website https://www.repository.cam.ac.uk/handle/1810/283491. eQTL datasets analyzed during the current study are available at GTEx Portal (https://www.gtexportal.org/home/datasets), BIOSQTL browser (https://molgenis26.gcc.rug.nl/downloads/biosqtlbrowser/) and eQTLGen consortium website (https://www.eqtlgen.org/cis-eqtls.html). Gene expression data used in this study are available at GEO database (https://www.ncbi.nlm.nih.gov/geo/).
