# Supplementary material for: Identifying novel genetic loci associated with polycystic ovary syndrome based on its shared genetic architecture with type 2 diabetes
Source: Front Genet. 2022 Aug 29;13:905716. doi: 10.3389/fgene.2022.905716 (PMC9464923; doi:10.3389/fgene.2022.905716)
Supplement: Supplementary file 1 [file Table2.docx]

Supplementary Material

# Participants

The genome-wide association study (GWAS) summary statistics of polycystic ovary syndrome (PCOS) were obtained from a hitherto largest GWAS (https://doi.org/10.17863/CAM.27720)(Day et al., 2018). The original study consisted of 10,074 PCOS cases and 103,164 controls from a total of 7 studies. The cases were diagnosed according to the National Institutes of Health (NIH) criteria, Rotterdam criteria, or self-report. NIH criteria required biochemical and/or clinical signs of hyperandrogenism (HA) and irregular menstruation due to ovulatory dysfunction (OD)(Zawadzki et al., 1992), while Rotterdam criteria include at least two of the following three traits: HA, OD, and polycystic ovary morphology (PCOM)(ESHRE and Group, 2004). However, the PCOS samples we gained consisted of 5209 cases and 32055 controls, excluding the self-report sample from the 23andMe database(n=87,943) due to data availability. Case status was defined by the NIH (2,540 cases and 15,020 controls) or Rotterdam Criteria (2,669 cases and 17,035 controls). All participants in PCOS GWASs were of European ancestry. A systematic review of data collection methods and procedures in each cohort was used by researchers to verify the quality of the phenotypic data, and only cohorts that reached these criteria were included. Next, cohort-level data has undergone stringent quality control procedures, including filtering out single nucleotide polymorphisms SNPs with high missingness, significant deviation from Hardy-Weinberg equilibrium, removing samples with low genotype call rate, and sex mismatch(Day et al., 2018). Principle components analysis was performed at each cohort to adjust population stratification. Using IMPUTE2 based on the 1000 Genomes Project and HapMap Project, genotype data were imputed. The SNP genotype data for each included cohort were individually filtered by minor allele frequency (MAF) >0.01 and an imputation quality metric, R^2^ > 0.3 or proper info > 0.4 for MACH and IMPUTE2, respectively. Further, association analysis was employed separately for each study. Finally, combined effects and P-values of genetic variants across these cohorts were generated by a fixed-effect inverse-weighted-variance meta-analysis using GWAMA or METAL software(Willer et al., 2010; Schork et al., 2013).

The Type 2 diabetes(T2D) GWAS summary statistics were obtained from the largest and most recently published GWAS meta-analysis in the DIAGRAM consortium (http://diagram-consortium.org/)(Mahajan et al., 2018). The study comprised a total of 74,124 cases and 824,006 controls from 32 cohorts in Europe. All participants were white Europeans. Cases were required to meet inclusive T2D diagnoses, such as self-reported history, diagnostic fasting glucose or HbA1c levels, hospital discharge diagnosis, use of oral diabetes medication, etc. stringent quality control procedures of sample and variant were performed within each study. Briefly, Variants were kept if (1) allele frequencies differed from the Haplotype Reference Consortium (HRC) reference panel by less than 20%(McCarthy et al., 2016) ;(2) MAF was in the range from 1% to 40%. Genotype data were imputed based on the HRC reference panel or 30440 Icelandic whole-genome sequences(Jónsson et al., 2017);(3)minor allele count >5(combined cases and controls);(4) imputation quality metric, R^2^ > 0.3 (miniMAC) or proper info > 0.4 (IMPUTE4);(5)standard error of allelic log OR < 10. For details on data processing, please see the original publication (Mahajan et al., 2018). Association analyses were performed for SNPs within each study, with and without adjustment for BMI, under an additive model of effect allele. Further, using METAL software, the association summary statistics were gathered to perform a fixed-effects meta-analysis with the inverse-variance weighting of log (ORs). According to the result of the genomic control factor, the BMI-unadjusted meta-analysis was corrected for residual inflation, while the BMI-adjusted meta-analysis did not need additional adjustment.

# MIXeR analysis

We calculated a z-score for each SNP from GWAS summary statistics of two traits. To quantify genetic overlap or shared genetic effects between traits, we applied a MiXeR modeling framework(https://github.com/precimed/mixer)(Frei et al., 2019). MiXeR method is a direct extension of cross-trait LD score regression which relaxes the infinitesimal assumption. Considering a simple additive model of effect alleles, without the influence of gene-environment interaction, epistasis, and dominance, we built a univariate causal mixture model for a single trait. Next, a bivariate causal mixture model was built between both traits under the assumption that all non-null variants follow the concordant distributions of effect size. Three Scenarios on the relationship between variants and traits were assumed:(1) some variants affect both traits;(2) some variants affect one trait but not the other;(3) Most variants do not affect either trait. $\text{β}_{\text{1j}}$ and $\text{β}_{\text{2j}}$, the effect size of genetic variant $j$ on the two traits GWAS, were modeled together as a mixture of four bivariate Gaussian components. However, some factors may influence the result of the estimated effect, such as linkage disequilibrium structure between genetic variants, limited sample size, and cryptic relatedness. To disentangle these effects, Observed GWAS signed test statistics can be deduced as：

$$\begin{aligned} \left( z_{1j},z_{2j} \right)\sim\left( \delta_{1j},\delta_{2j} \right)+N\left( \left( 0,0 \right),\left[ \begin{matrix} \sigma_{01}^{2} & \rho_{0}\sigma_{01}\sigma_{02} \\ \rho_{0}\sigma_{01}\sigma_{02} & \sigma_{02}^{2} \end{matrix} \right] \right)\#\left( 1 \right) \end{aligned}$$

$$\begin{aligned} \delta_{\star j}=\sqrt{N_{\star j}}\sum_{i} \sqrt{H_{j}}r_{ij}\beta_{\star j}\#\left( 2 \right) \end{aligned}$$

where $r_{ij}$ is the allelic correlation between two different variants, representing the effects of LD structure; $H_{j}$ is the heterozygosity; $N_{\star j}$ is the number of samples genotyped per variant of trait1 or trait2. $\sigma_{01}^{2}$, $\sigma_{02}^{2}$ and $\rho_{0}$are the variance distortion parameters. We then fit the model by direct optimization of weighted log-likelihood using Nelder-Mead Simplex Method(Lagarias et al., 1998). Specifically, we first fitted the univariate model separately for a single trait and find univariate parameters ($\pi_{1}$, $\sigma_{1}$*,* $\sigma_{01}$.) by using “fast model” under specific parameters constraint; secondly, “full model” and unconstrained optimization are used together to estimate $\pi_{1}$, $\sigma_{1}$*,* $\sigma_{01}$. we repeated the above steps for trait2 to find $\pi_{2}$, $\sigma_{2}$*,* $\sigma_{02}$;thirdly, in the bivariate model, we use the “fast model” to find $r_{g}$ and $\rho_{0}$; finally, we define the parameters of shared component ($\pi_{12}$, $\rho_{12}$) constraining all above estimated parameters. $r_{g}$,a genome-wide genetic correlation, was also computed using MIXeR tool：

$$\begin{aligned} r_{g}= \frac{\rho_{12}\pi_{12}}{\sqrt{\pi_{1}^{u}\pi_{2}^{u}}} \#\left( 3 \right) \end{aligned}$$

Where $\pi_{1}^{u}= \pi_{1}+\pi_{12}$ and $\pi_{2}^{u}= \pi_{2}+\pi_{12}$.After fitting model, we can evaluate the estimated number of quantity variants of shared components which explains 90% of SNP heritability in each trait, and the dice coefficient, which is an overall measure of polygenic overlap between traits, and then visualize them with a Venn diagram.

# Cross-trait linkage disequilibrium score regression (LDSC) method

In order to identify genetic correlations between PCOS and T2D with or without adjustment of BMI, we used the cross-trait LDSC method through the LDSC tool(Bulik-Sullivan et al., 2015)( http://www.github.com/bulik/ldsc). The LDSC tool estimates the bivariate genetic correlations of a phenotype with another trait using individual SNP allele effect sizes and the average LD in a region. In this study, those with P values < 0.05 should be considered significant surviving Bonferroni correction for multiple testing. Significantly, the definition of genetic correlation differs from polygenic overlap. Two traits have a potential pleiotropic relationship if many variants affect both, regardless of their allelic effect directions. Compared with polygenic overlap, the condition of genetic correlation is more stringent: to exhibit genetic correlation, the directions of effect must also be consistently aligned(Bulik-Sullivan et al., 2015).). Therefore, the LDSC method cannot deal with the situation that most shared genetic loci between two traits have opposite directions of effect. MiXeR can capture the shared genetic basis even when the effects are mixed and cancel each other out in correlation analyses(Frei et al., 2019).

# Data quality control and preprocessing

Prior to the exploration of the pleiotropic loci, 7272272 variants were defined across PCOS and T2D traits. Ambiguous SNPs were filtered out first. We then removed the human major histocompatibility complex (MHC) region (hg19 as chr6: 25119106- 33854733) and 8p23.1 inversion (hg19 as chr8: 7200000-12500000) because of the high Linkage disequilibrium (LD) between genes of MHC and the high frequency of the inverted allele in 8p23.1 inversion leading to bias in estimating FDR(Bosch et al., 2009; Trowsdale and Knight, 2013). The remaining 6136626 SNPs were used for further analyses. All P-values were adjusted by genomic control inflation factor $\lambda_{GC}$ which is calculated by leveraging only intergenic SNPs, because genomic control for intergenic SNPs is most likely to minimize the inflation due to polygenic effects and provide a robust estimate of null effects, while genomic control for all SNPs may lead to deflation due to the over-correction of test statistics(Schork et al., 2013).

# Conditional quantile-quantile (Q-Q) plots

The Q-Q plots compare the nominal probability distribution of p-values against an empirical distribution of p-values. Q-Q plot shows a linear trend concordant with the line y=x when all SNPs are null. In the usual Q-Q plot, the nominal p-values denoted by “$p$” were plotted as the y-ordinate, while the empirical p-values denoted by “$q$” were plotted as the x-ordinate. For T2D and PCOS, $-{log}_{10}p$ and $-{log}_{10}q$ were used instead of the initial p-value to accentuate the tail probabilities of the theoretical and empirical distributions. We applied conditional quantile-quantile (Q-Q) plots for one phenotype based on varying levels of association with another phenotype under the null hypothesis and vice versa to intuitively assess for pleiotropic enrichment. Specifically, we stratified the SNPs into subsets based on different nominal P-values of the primary phenotype associated with the secondary phenotype ($-{log}_{10}p>0$, $-{log}_{10}p$>1, $-{log}_{10}p>2$, $-{log}_{10}p>3$, corresponding to $p<1, p<0.1, p<0.01, p<0.001$, respectively). To assess for polygenic effects below the standard GWAS significance threshold, Q-Q plots on SNPs with nominal$-{log}_{10}p<7.3$ (corresponding to $p>5\times{10}^{-8}$) were focused on. Pleiotropic enrichment exists if the Q-Q curve was plotted as successive leftward deflections from the null distribution, corresponding to a larger proportion of SNPs with a nominal $-{log}_{10}p$ value greater than or equal to a given threshold. We constructed conditional Q-Q plots after random pruning averaged over 100 iterations to control for spurious enrichment. Random SNP in every LD block (defined by an $r^{2} >0.1$) was selected, and the empirical cumulative distribution function was computed using the corresponding p-values at each iteration. As well as QQ plot, condFDR and conjFDR analyses were performed after random pruning for all SNPs across 100 iterations by selecting one random SNP per linkage disequilibrium block (defined by LD $r^{2} >0.1$). For more detail, see original publications(Smeland et al., 2020)

# Conditional false discovery rate

To improve the detectability of risk variants associated with PCOS, we applied a conditional false discovery rate (condFDR) statistical method(https://github.com/precimed/pleiofdr)(Smeland et al., 2020). False discovery rate (FDR) is defined as the expected probability of incorrect rejections for a random SNP. The conditional FDR is an extension of FDR, defined as the posterior probability that a random SNP is null for the first phenotype given that the observed p-values for both phenotypes are as small as or smaller than the predefined p-values. It can be expressed as:

$$\begin{aligned} condFDR\left( p_{1},p_{2} \right)= \frac{\pi_{0}\left( p_{2} \right)p_{1}}{F\left( p_{1}\left| p_{2} \right. \right)}\#\left( 4 \right) \end{aligned}$$

where $p_{1}$ and $p_{2}$ are p-values of SNPs in the primary and the secondary phenotypes correspondingly; $\pi_{0}\left( p_{2} \right)$ is the proportion of null SNPs conditional on second phenotypes; $F\left( p_{1}\left| p_{2} \right. \right)$ is the conditional CDF for the first phenotype given that p-values for the second phenotype are as small as $p_{2}$ or smaller. We denote the condFDR for phenotype 1 give phenotype 2 as ${FDR}_{trait1\left| trait2 \right.}$, We also perform a conservative estimate of condFDR by setting $\pi_{0}\left( p_{2} \right)$equal to 1 and replacing $F\left( p_{1}\left| p_{2} \right. \right)$ with empirical conditional CDF. SNPs with a condFDR value less than 0.05 were considered statistically significantly associated with the primary phenotype.

# Conjunctional false discovery rate

Conjunctional false discovery rate (conjFDR) is used to further identify SNP that are associated with two phenotypes simultaneously，which is defined as the posterior probability that a random SNP is null for either phenotype or both simultaneously, given that the observed p-values for both phenotypes are as small as or smaller than the predefined p-values(https://github.com/precimed/pleiofdr)(O’Connell et al., 2019). Formally, the conjunctional FDR is given by:

$$\begin{aligned} conjFDR\left( p_{1},p_{2} \right)= \frac{\pi_{0}F_{0}\left( {p_{1},p}_{2} \right)}{F\left( p_{1},p_{2} \right)}+\frac{\pi_{1}F_{1}\left( {p_{1},p}_{2} \right)}{F\left( p_{1},p_{2} \right)}+\frac{\pi_{2}F_{2}\left( {p_{1},p}_{2} \right)}{F\left( p_{1},p_{2} \right)}\#\left( AUTONUM \backslash* Arabic \right) \end{aligned}$$

where $\pi_{0}$ is the priori fraction of null SNPs for both phenotypes simultaneously;$F_{0}\left( {p_{1},p}_{2} \right)$ is the joint null CDF;$\pi_{1}$ is the priori fraction of SNPs cumulative distribution function of SNPs non-null for phenotype 1 but null for phenotype 2; $F_{1}\left( {p_{1},p}_{2} \right)$ is the join3t CDF of these SNPs.$\pi_{2}$ and $F_{2}\left( {p_{1},p}_{2} \right)$ are similar to $\pi_{1}$ and $F_{1}\left( {p_{1},p}_{2} \right)$ but the SNPs is null for phenotype 1 but non-null for phenotype 2; $F\left( p_{1},p_{2} \right)$ is the joint overall mixture CDF for all SNPs of both phenotypes. We signify conjFDR of for phenotype 1 and phenotype 2 as ${FDR}_{trait1\&trait2}$. SNPs with a conjFDR value less than 0.05 was deemed as the shared variants.

# Definition of novel genetic variants

SNPs having a condFDR or conjFDR less than 0.05 and at LD r^2^<0.1 with each other were considered as lead SNPs. Next, we aim to identify the potential risk loci of PCOS. Specifically, we first single out the lead SNP that were even less closely related. SNPs having a condFDR or conjFDR < 0.05 and independent of other SNPs at LD $r^{2}<0.6$ were considered as significant independent SNPs. Those SNPs independent of other SNPs at LD $r^{2}<0.1$ were then selected as lead SNPs. The border for a genomic locus was defined as a region containing all candidate SNPs in LD ($r^{2}\geq0.6$) with a lead SNP. Candidate SNPs were merged into a genomic locus if the distances between them were less than 250Kb. We finally found 11 lead SNPs of ${FDR}_{PCOS\left| T2DM \right.}$ and 6 lead SNPs of ${FDR}_{PCOS\&2DM}$. To determine if these loci were novel, we first checked if P-values for lead SNPs were less than $5\times{10}^{-8}$ in the original PCOS GWAS and excluded those with P value less than $5\times{10}^{-8}$. Secondly, we performed a search of GWAS catalog, which is an online database with publicly available resources of all human genome-wide association studies(MacArthur et al., 2016). we queried the lead SNPs using the term (“SNP name”) to identify those associated with PCOS at genome-wide significance in other GWAS datasets. We also computed $r^{2}$between each lead SNP in this study and reported SNPs and then defined novel loci as the lead SNPs having independent of all reported SNPs at LD $r^{2}<0.6$. Finally, we evaluated the direction of allelic effects of the shared genetic variants by comparing the T2D z-scores against PCOS traits z-scores.

# Functional annotation

Functional mapping and annotation (FUMA) protocol, an online platform(<http://fuma.ctglab.nl/>), was applied to annotate and prioritize independent genomic loci using information from 18 biological data repositories and tools. Novel lead SNPs and SNPs having an LD (r^2^>≥0.6) with one of the lead SNPs ( in the same genomic region) were annotated with Combined Annotation Dependent Depletion (CADD) scores(Kircher et al., 2014), Regulome DB scores(Boyle et al., 2012), and chromatin states(Roadmap Epigenomics Consortium et al., 2015).

The CADD is a general framework for integrating the genetic variation with a diverse set of genome annotations and scoring any possible human single-nucleotide variant (SNV), the most genetic variants in the human genome, or small insertion/deletion (indel) event(Kircher et al., 2014). CADD evaluates the deleteriousness of genetic variants by integrating 63 functional annotations using a support vector machine(Kircher et al., 2014). The higher the CADD score, the more deleterious. A CADD score above 12.37 is the threshold to be potentially pathogenic(Kircher et al., 2014).

The Regulome DB is an integrated database which can classify genetic variant using a heuristic scoring system based on functional confidence of a variant(Boyle et al., 2012). Regulome DB score the SNPs based on kinds of information from known classes of genomic elements such as promoters, enhancers, transcription start sites, and transcription factor (TF) binding motifs, ranging from 1 to 7. Lower scores indicate increasing confidence that a genetic variant is located in a functional location and likely results in a functional consequence (i.e., alteration of TF binding and a gene regulatory effect) .Scores are as follows: 1a= eQTL + TF binding + matched TF motif + matched DNase Footprint + DNase peak; 1b= eQTL + TF binding + any motif + DNase Footprint + DNase peak; 1c= eQTL + TF binding + matched TF motif + DNase peak; 1d= eQTL + TF binding + any motif + DNase peak; 1e= eQTL + TF binding + matched TF motif; 1f= eQTL + TF binding / DNase peakeQTL + TF binding / DNase peak; 2a= TF binding + matched TF motif + matched DNase Footprint + DNase peak; 2b= TF binding + any motif + DNase Footprint + DNase peak; 2c=TF binding + matched TF motif + DNase peak; 3a= TF binding + any motif + DNase peak; 3b= TF binding + matched TF motif; 4= TF binding + DNase peak; 5= TF binding or DNase peak; 6= Motif hit; 7=other(Boyle et al., 2012). Category 1(1a-1f) represents that selected SNP is likely to affect binding and linking to expression of a gene target; Category (2a-2c) represents that selected SNP likely to affect binding; Category 3(3a-3b) represents that selected SNP is less likely to affect binding; Categories 4 to 7 represents insufficient evidence of the selected SNP actually disrupting the site of binding. SNPs with Regulome DB score $\leq2$ were defined as SNPs being functional.

The chromatin state represents the accessibility of genomic regions (every 200bp) with 15 categorical states predicted by the hidden Markov model consisting of 8 active states and 7 repressed states, based on 5 chromatin marks for 127 epigenomes in the Roadmap Epigenomics(Roadmap Epigenomics Consortium et al., 2015). A lower state indicates higher accessibility, with states 1-7 referring to open chromatin states. We annotated the minimum chromatin state across tissues to SNPs. The 15-core chromatin states as suggested by Roadmap are as follows: 1=Active Transcription Start Site (TSS); 2=Flanking Active TSS; 3=Transcription at gene 5’ and 3’; 4=Strong transcription; 5= Weak Transcription; 6=Genic enhancers; 7=Enhancers; 8=Zinc finger genes & repeats; 9=Heterochromatic; 10=Bivalent/Poised TSS; 11=Flanking Bivalent/Poised TSS/Enh; 12=Bivalent Enhancer; 13=Repressed PolyComb; 14=Weak Repressed PolyComb; 15=Quiescent/Low.

# Expression quantitative trait loci (eQTL) analysis

All expression patterns of the novel loci were investigated to explore the correlations between genotype and tissue-specific gene expression levels. We examined the eQTL functionality for lead SNPs using the publicly available data from the Genotype-Tissue Expression (GTEx) Project (Lonsdale et al., 2013), BIOS Consortium (BIOSQTL)(Zhernakova et al., 2017), and eQTLGen Consortium (eQTLGen)(Võsa et al., 2021). In the GTEx project (https://gtexportal.org, Release V8), expression data in 49 tissues from 838 donors was profiled on both Affymetrix Human Gene 1.1 ST Expression Array and Illumina TrueSeq RNA sequencing, and individuals were genotyped using the Illumina OMNII 5M SNP Array. The FastQTL method was used to generate the candidate QTLs for tissues having more than 70 donors. Beta distribution-adjusted empirical p-values from FastQTL were used to calculate q-values, and an FDR threshold of 0.05 was applied to identify genes with a significant eQTL (Storey and Tibshirani, 2003). We used 49 tissue eQTL data from GTEx-V8 to define the eQTL functionality of identified lead SNPs.

Expression data from BIOSQTL and eQTLGen were further applied to validate the eQTL association identified in the GTEx project. BIOSQTL(Zhernakova et al., 2017) comprised 2116 healthy adults from four cohorts. Individual-level whole blood expression was measured using Illumina TruSeq version 2 and genotype data were imputed per cohort with IMPUTE2 using the GoNL reference panel(V5)(Deelen et al., 2014), resulting in 9333740 SNPs passing quality control. Finally, BIOSQTL determines independent QTL effects by using stepwise regression and replicating the result of eQTL in the external expression dataset. In addition to whole blood eQTL data from BIOSQTL, we also assessed the replicability of eQTL associations identified in the GTEx dataset using eQTLGen data(Võsa et al., 2021). The eQTLGen dataset consists of 31684 whole-blood and Peripheral blood mononuclear cell (PBMC) samples from 37 datasets, 25,482 (80.4%) of the samples were whole-blood samples, and 6,202 (19.6%) were PBMC samples. Gene expression levels of samples were profiled by Illumina, Affymetrix U219, and Affymetrix Hu-Ex version 1.0 ST expression arrays and by RNA-seq, and each individual was genotyped on 1000 Genomes phase 1 version 3 (1000G p1v3) or a newer reference panel. Finally, whole-genome eQTL data of 31684 samples were further meta-analyzed. For full details, see the original publication(Lonsdale et al., 2013; Zhernakova et al., 2017; Võsa et al., 2021).

# Differential expression analysis

To determine whether the novel loci identified by condFDR or conjFDR analysis were differentially expressed in PCOS cases, we used publicly available gene expression data obtained from Gene Expression Omnibus(https://www.ncbi.nlm.nih.gov/geo/). Linear models for Microarray Data were implemented for four datasets (GSE10946(Kenigsberg et al., 2009), GSE98595(Ferrero et al., 2018), GSE8157(Skov et al., 2008), GSE48301(Piltonen et al., 2013)) by using an R package ‘LIMMA’ from Bioconductor(https://bioconductor.org/packages/release/bioc/html/limma.html)(Ritchie et al., 2015). Those genes with P-values less than 0.05 were deemed as potentially and nominally differential expression genes. The brief descriptions of the above GEO datasets are as follows:

The study samples of GSE10946 consisted of cumulus cells collected from 12 patients with lean (body mass index (BMI) < 24) or overweight-obese PCOS (BMI > 27) and 13 matching controls(Kenigsberg et al., 2009). All the participants included in the study were women undergoing in-vitro fertilization with intra-cytoplasmic sperm injection and the PCOS patients were diagnosed according to the Rotterdam criteria after excluding secondary causes. Cumulus cell RNA samples were analyzed for transcriptome analysis using Affymetrix GeneChip Human Genome U133 Plus 2.0, which includes 54,675 probes, with one sample per array.

The PCOS patients from GSE98595 were determined by Rotterdam criteria and their lutein granulosa cells were obtained at the time of oocyte retrieval from women aged 25 to 32 years(Ferrero et al., 2018). The study also isolated the lutein granulosa cells from follicular fluid of women without PCOS (controls). RNA-level expressions were measured using Affymetrix Human Gene 1.0 ST Array, including 33297 probes, with one sample per array.

We selected the skeletal muscle samples of 10 PCOS patients and 13 controls from GSE8157 datasets(Ferrero et al., 2018). Women with PCOS were diagnosed as follows:(1) irregular periods with cycle length >35 days during the last year;(2) free testosterone level above reference interval (>0.035 nmol/l); (3) and/or hirsutism (total Ferriman-Gallwey score>7). All the patients accepted to withdraw oral contraceptives >3 months before evaluation and consented to use barrier contraception combined with spermicidal cream during the study period. Control subjects had regular menses, normal glucose tolerance, and no family history of diabetes. The study excluded women with diabetes (fasting plasma glucose≥7.0 mmol/l), hypertension, elevated liver enzyme levels, adrenal enzyme defects, hyperprolactinemia, and hypothyroidism. In addition, participants were excluded if they were pregnant. RNA samples were analyzed for transcriptome analysis using Affymetrix GeneChip Human Genome U133 Plus 2.0.

The study samples of GSE48301 comprised endometrial cells collected from 6 patients with PCOS (diagnosed as the existence of HA, PCOM, and/or OD) and 6 controls (Piltonen et al., 2013). All controls reported the menstrual cycles with regular intervals (25–35 d) and no clinical evidence of having PCOS. All participants were excluded if they were pregnant and exposed to hormonal medications for at least 2 months. Gene expressions were analyzed for transcriptome analysis using Affymetrix Human Gene 1.0 ST Array. Further details about sample selection, RNA measurement, quality control, and statistical analysis can be found in the original publication.

# Reference

Bosch, N., Morell, M., Ponsa, I., Mercader, J.M., Armengol, L., and Estivill, X. (2009). Nucleotide, cytogenetic and expression impact of the human chromosome 8p23.1 inversion polymorphism. *PloS one* 4(12)**,** e8269-e8269. doi: 10.1371/journal.pone.0008269.

Boyle, A.P., Hong, E.L., Hariharan, M., Cheng, Y., Schaub, M.A., Kasowski, M., et al. (2012). Annotation of functional variation in personal genomes using RegulomeDB. *Genome Research* 22(9)**,** 1790-1797. doi: 10.1101/gr.137323.112.

Bulik-Sullivan, B., Finucane, H.K., Anttila, V., Gusev, A., Day, F.R., Loh, P.R., et al. (2015). An atlas of genetic correlations across human diseases and traits. *Nat Genet* 47(11)**,** 1236-1241. doi: 10.1038/ng.3406.

Day, F., Karaderi, T., Jones, M.R., Meun, C., He, C., Drong, A., et al. (2018). Large-scale genome-wide meta-analysis of polycystic ovary syndrome suggests shared genetic architecture for different diagnosis criteria. *PLOS Genetics* 14(12)**,** e1007813.

Deelen, P., Menelaou, A., van Leeuwen, E.M., Kanterakis, A., van Dijk, F., Medina-Gomez, C., et al. (2014). Improved imputation quality of low-frequency and rare variants in European samples using the ‘Genome of The Netherlands’. *European Journal of Human Genetics* 22(11)**,** 1321-1326. doi: 10.1038/ejhg.2014.19.

Efron, B. (2007). Size, power and false discovery rates. *Annals of Statistics* 35(4)**,** 1351-1377, 1327.

Efron, B. (2012). *Large-scale inference: empirical Bayes methods for estimation, testing, and prediction.* Cambridge University Press.

ESHRE, T.R., and Group, A.-S.P.C.W. (2004). Revised 2003 consensus on diagnostic criteria and long-term health risks related to polycystic ovary syndrome. *Fertility and Sterility* 81(1)**,** 19-25.

Ferrero, H., Díaz-Gimeno, P., Sebastián-León, P., Faus, A., Gómez, R., and Pellicer, A. (2018). Dysregulated genes and their functional pathways in luteinized granulosa cells from PCOS patients after cabergoline treatment. *Reproduction* 155(4)**,** 373-381. doi: 10.1530/REP-18-0027.

Frei, O., Holland, D., Smeland, O.B., Shadrin, A.A., Fan, C.C., Maeland, S., et al. (2019). Bivariate causal mixture model quantifies polygenic overlap between complex traits beyond genetic correlation. *Nature Communications* 10(1)**,** 2417-2417. doi: 10.1038/s41467-019-10310-0.

Jónsson, H., Sulem, P., Kehr, B., Kristmundsdottir, S., Zink, F., Hjartarson, E., et al. (2017). Whole genome characterization of sequence diversity of 15,220 Icelanders. *Scientific Data* 4(1)**,** 170115. doi: 10.1038/sdata.2017.115.

Kenigsberg, S., Bentov, Y., Chalifa-Caspi, V., Potashnik, G., Ofir, R., and Birk, O.S. (2009). Gene expression microarray profiles of cumulus cells in lean and overweight-obese polycystic ovary syndrome patients. *Molecular human reproduction* 15(2)**,** 89-103. doi: 10.1093/molehr/gan082.

Kircher, M., Witten, D.M., Jain, P., O'Roak, B.J., Cooper, G.M., and Shendure, J. (2014). A general framework for estimating the relative pathogenicity of human genetic variants. *Nature Genetics* 46(3)**,** 310-315. doi: 10.1038/ng.2892.

Lagarias, J.C., Reeds, J.A., Wright, M.H., and Wright, P.E. (1998). Convergence Properties of the Nelder--Mead Simplex Method in Low Dimensions. *SIAM Journal on Optimization* 9(1)**,** 112-147. doi: 10.1137/s1052623496303470.

Lonsdale, J., Salvatore, M., Phillips, R., Garcia, F., Young, N., Foster, B., et al. (2013). The Genotype-Tissue Expression (GTEx) project. *Nature Genetics* 45(6)**,** 580-585. doi: 10.1038/ng.2653.

MacArthur, J., Bowler, E., Cerezo, M., Gil, L., Hall, P., Hastings, E., et al. (2016). The new NHGRI-EBI Catalog of published genome-wide association studies (GWAS Catalog). *Nucleic Acids Research* 45(D1)**,** D896-D901. doi: 10.1093/nar/gkw1133 %J Nucleic Acids Research.

Mahajan, A., Taliun, D., Thurner, M., Robertson, N.R., Torres, J.M., Rayner, N.W., et al. (2018). Fine-mapping type 2 diabetes loci to single-variant resolution using high-density imputation and islet-specific epigenome maps. *Nature Genetics* 50(11)**,** 1505-1513. doi: 10.1038/s41588-018-0241-6.

McCarthy, S., Das, S., Kretzschmar, W., Delaneau, O., Wood, A.R., Teumer, A., et al. (2016). A reference panel of 64,976 haplotypes for genotype imputation. *Nature Genetics* 48(10)**,** 1279-1283. doi: 10.1038/ng.3643.

Nichols, T., Brett, M., Andersson, J., Wager, T., and Poline, J.-B. (2005). Valid conjunction inference with the minimum statistic. *NeuroImage* 25(3)**,** 653-660. doi: <https://doi.org/10.1016/j.neuroimage.2004.12.005>.

O’Connell, K.S., Shadrin, A., Bahrami, S., Smeland, O.B., Bettella, F., Frei, O., et al. (2019). Identification of genetic overlap and novel risk loci for attention-deficit/hyperactivity disorder and bipolar disorder. *Molecular Psychiatry*. doi: 10.1038/s41380-019-0613-z.

Piltonen, T.T., Chen, J., Erikson, D.W., Spitzer, T.L.B., Barragan, F., Rabban, J.T., et al. (2013). Mesenchymal Stem/Progenitors and Other Endometrial Cell Types From Women With Polycystic Ovary Syndrome (PCOS) Display Inflammatory and Oncogenic Potential. *Journal of Clinical Endocrinology and Metabolism* 98(9)**,** 3765-3775. doi: 10.1210/jc.2013-1923.

Ritchie, M.E., Phipson, B., Wu, D., Hu, Y., Law, C.W., Shi, W., et al. (2015). limma powers differential expression analyses for RNA-sequencing and microarray studies. *Nucleic Acids Research* 43(7)**,** e47-e47. doi: 10.1093/nar/gkv007 %J Nucleic Acids Research.

Roadmap Epigenomics Consortium, E.C., Kundaje, A., Meuleman, W., Ernst, J., Bilenky, M., Yen, A., et al. (2015). Integrative analysis of 111 reference human epigenomes. *Nature* 518(7539)**,** 317-329. doi: 10.1038/nature14248.

Schork, A.J., Thompson, W.K., Pham, P., Torkamani, A., Roddey, J.C., Sullivan, P.F., et al. (2013). All SNPs are not created equal: genome-wide association studies reveal a consistent pattern of enrichment among functionally annotated SNPs. *PLoS genetics* 9(4)**,** e1003449-e1003449. doi: 10.1371/journal.pgen.1003449.

Skov, V., Glintborg, D., Knudsen, S., Tan, Q., Jensen, T., Kruse, T.A., et al. (2008). Pioglitazone enhances mitochondrial biogenesis and ribosomal protein biosynthesis in skeletal muscle in polycystic ovary syndrome. *PloS one* 3(6)**,** e2466-e2466. doi: 10.1371/journal.pone.0002466.

Smeland, O.B., Frei, O., Shadrin, A., O’Connell, K., Fan, C.-C., Bahrami, S., et al. (2020). Discovery of shared genomic loci using the conditional false discovery rate approach. *Human Genetics* 139(1)**,** 85-94. doi: 10.1007/s00439-019-02060-2.

Storey, J.D., and Tibshirani, R. (2003). Statistical significance for genomewide studies. *Proceedings of the National Academy of Sciences, USA* 100(16)**,** 9440. doi: 10.1073/pnas.1530509100.

Trowsdale, J., and Knight, J.C. (2013). Major histocompatibility complex genomics and human disease. *Annual Review of Genomics & Human Genetics* 14**,** 301-323. doi: 10.1146/annurev-genom-091212-153455.

Võsa, U., Claringbould, A., Westra, H.-J., Bonder, M.J., Deelen, P., Zeng, B., et al. (2021). Large-scale cis- and trans-eQTL analyses identify thousands of genetic loci and polygenic scores that regulate blood gene expression. *Nature Genetics* 53(9)**,** 1300-1310. doi: 10.1038/s41588-021-00913-z.

Willer, C.J., Li, Y., and Abecasis, G.R. (2010). METAL: fast and efficient meta-analysis of genomewide association scans. *Bioinformatics* 26(17)**,** 2190-2191. doi: 10.1093/bioinformatics/btq340.

Zawadzki, J., Dunaif, A., Givens, J., Haseltine, F., and Merriam, G. (1992). "Current issues in endocrinology and metabolism: polycystic ovary syndrome". Cambridge, MA: Blackwell Scientific Publications).

Zhernakova, D.V., Deelen, P., Vermaat, M., van Iterson, M., van Galen, M., Arindrarto, W., et al. (2017). Identification of context-dependent expression quantitative trait loci in whole blood. *Nature Genetics* 49(1)**,** 139-145. doi: 10.1038/ng.3737.
